# Is endoscopic radiofrequency ablation safe and effective for treating rare neuroendocrine tumors of the minor papilla?

**DOI:** 10.1055/a-2610-2477

**Published:** 2025-06-18

**Authors:** Rong Wang, Zian Su, Hengwei Zhang, Jinduo Zhang, Ping Yue, Wenbo Meng, Xun Li

**Affiliations:** 1 The First School of Clinical Medicine, Lanzhou University, Lanzhou, China; 2 Department of General Surgery, The First Hospital of Lanzhou University, Lanzhou, China; 3 Hepatopancreatobiliary Surgery Institute of Gansu Province, Lanzhou, China

A 62-year-old man had asymptomatic pancreatic duct dilation for 4 years without further diagnosis. Subsequently, during a computed tomography scan following lung cancer surgery, a mass with abnormal enhancement was detected incidentally at the major duodenal papilla. Magnetic resonance imaging revealed a nodule (approximately 12 × 9 mm) with an abnormal signal and a dilated pancreatic duct ( Fig. 1 ). Gastroscopy revealed an ulcer at the minor duodenal papilla. Biopsy pathology results suggested a neuroendocrine tumor ( Fig. 2 ).

**Fig. 1:**
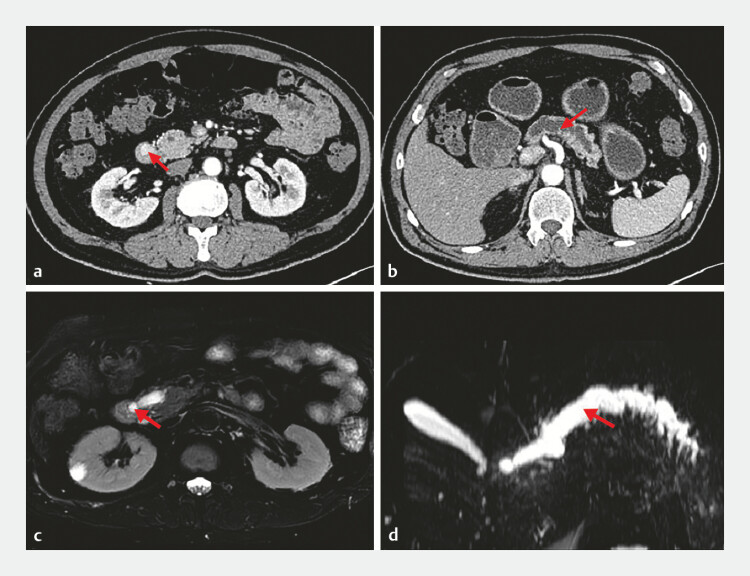
Imaging studies. **a, b** Computed tomography imaging revealed abnormal enhancement of the major duodenal papilla with significant dilation of the pancreatic duct (arrow). **c, d** Magnetic resonance imaging revealed an abnormal signal nodule in the major papilla of the duodenum, with significant dilation of the pancreatic duct (arrow).

**Fig. 2:**
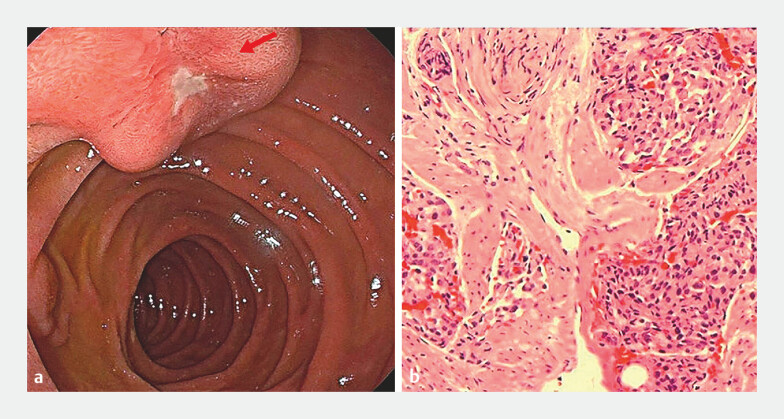
Gastroscopy and pathology findings. **a** Gastroscopy revealed a duodenal minor papillary ulcer (arrow). **b** Biopsy pathology revealed mild-to-moderate atypical hyperplasia of the glandular epithelium.

The patient chose to undergo endoscopic retrograde cholangiopancreatography (ERCP)-guided radiofrequency ablation (RFA) rather than surgery. During the ERCP procedure, the tumor was found to be located at the duodenal minor papilla rather than the major papilla. After failure of pancreatic duct cannulation through the major papilla, the guidewire entered the biliary duct, and fluoroscopy revealed a bile duct diameter of 3 mm ( Fig. 3 ). Successful cannulation through the minor papilla was subsequently achieved with a 0.025-inch straight-tip guidewire. Fluoroscopy revealed distal pancreatic duct dilation and proximal stenosis. Endoscopic RFA (Boston Scientific, Besançon, France) was then performed at 10 W for 90 seconds ( Fig. 4 ). A pancreatic duct stent (Cook Medical, Limerick, Ireland) was placed at the minor papilla, and a bile duct stent (Boston Scientific, Spencer, Indiana, USA) was placed at the major papilla ( Video 1 ). The patient did not experience any postoperative complications.

**Fig. 3:**
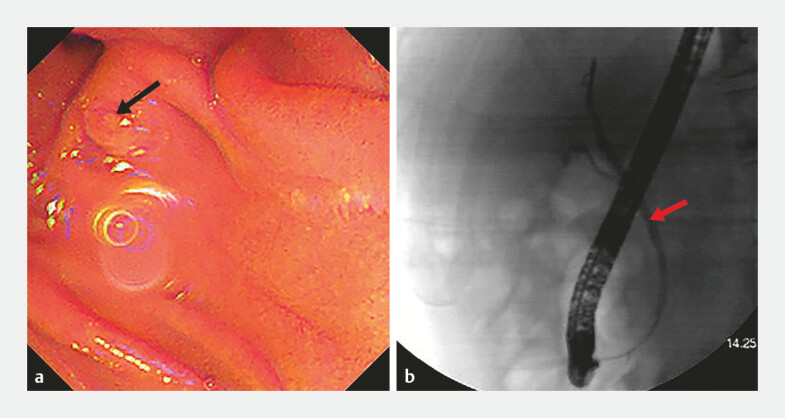
Endoscopic retrograde cholangiopancreatography and fluoroscopy. **a** The major duodenal papilla (arrow) and periampullary diverticulum. **b** Fluoroscopy at the major duodenal papilla revealed a bile duct diameter of approximately 3 mm (arrow).

**Fig. 4:**
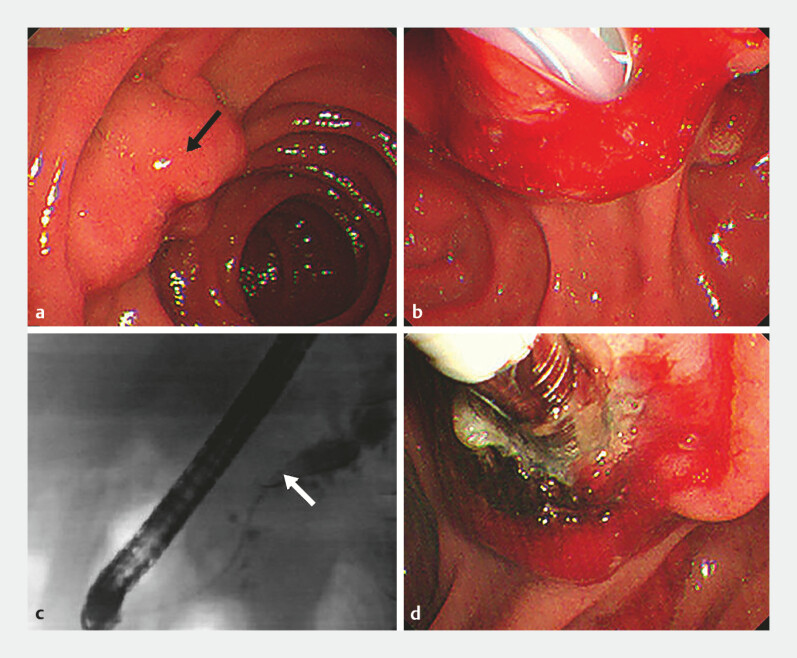
Cannulation and radiofrequency ablation (RFA). **a** Minor papillary neoplasia (arrow). **b** Successful cannulation of the minor papilla. **c** Angiography of the minor papilla (arrow). **d** Endoscopic RFA of the minor papillary neoplasia.

Endoscopic retrograde cholangiopancreatography and radiofrequency ablation procedures.Video 1

At the 6-month follow-up ERCP, the tumor size had reduced ( Fig. 5 ), and additional RFA was performed without any post-ERCP complications.

**Fig. 5:**
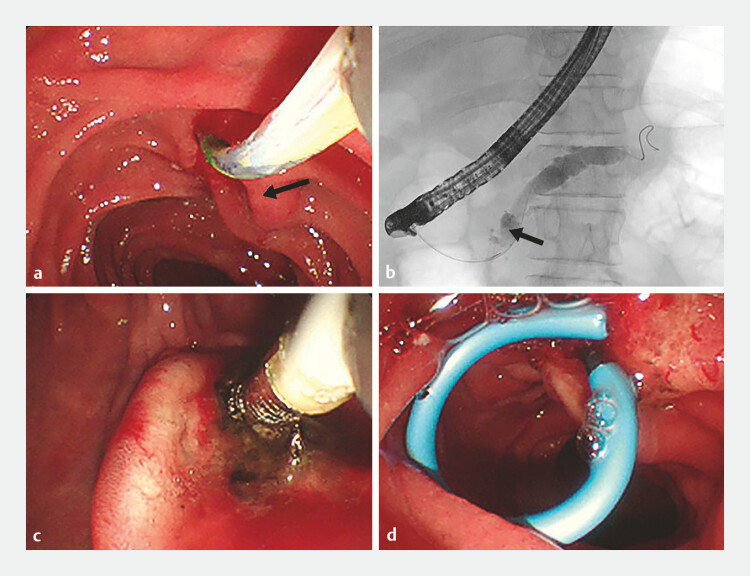
Follow-up visit at 6 months. **a** The minor papillary neoplasia (arrow) had reduced in size, and the bile duct stent at the major papilla had detached. **b** Pancreatic fluoroscopy revealed relief of pancreatic duct narrowing (arrow) at the pancreatic head and a reduction in the distal pancreatic duct diameter. **c** Endoscopic radiofrequency ablation of the minor papillary neoplasia was repeated. **d** A new pancreatic duct stent was placed.

Ampullary neoplasms are uncommon, accounting for less than 0.5% of all gastrointestinal neoplasms, but they can often be malignant 1 , and minor papillary neoplasia tumors are even rarer. RFA has been performed widely in the treatment of cholangiocarcinoma and periampullary tumors, and its safety and efficacy have been confirmed 2 . However, to our knowledge, there have been no reports of RFA for minor papillary neoplasia in patients with pancreas divisum. Minor papillary neoplasias are rare, and cannulation of the minor papilla is challenging 3 . This case confirms the feasibility and safety of the use of RFA in the treatment of minor papillary neoplasias, suggesting that this method can be implemented in similar patients.

Endoscopy_UCTN_Code_CCL_1AZ_2AM

## Notes

Endoscopy E-Videos https://eref.thieme.de/e-videos
*E-Videos* is an open access online section of the journal *Endoscopy* , reporting on interesting cases and new techniques in gastroenterological endoscopy. All papers include a high-quality video and are published with a Creative Commons CC-BY license. Endoscopy E-Videos qualify for HINARI discounts and waivers and eligibility is automatically checked during the submission process. We grant 100% waivers to articles whose corresponding authors are based in Group A countries and 50% waivers to those who are based in Group B countries as classified by Research4Life (see: https://www.research4life.org/access/eligibility/ ). This section has its own submission website at https://mc.manuscriptcentral.com/e-videos .
